# Butyrate Enhances Desensitization Induced by Oral Immunotherapy in Cow's Milk Allergic Mice

**DOI:** 10.1155/2019/9062537

**Published:** 2019-01-16

**Authors:** Marlotte M. Vonk, Bart R. J. Blokhuis, Mara A. P. Diks, Laura Wagenaar, Joost J. Smit, Raymond H. H. Pieters, Johan Garssen, Léon M. J. Knippels, Betty C. A. M. van Esch

**Affiliations:** ^1^Department of Pharmacology, Utrecht Institute for Pharmaceutical Sciences, Faculty of Science, Utrecht University, Universiteitsweg 99, 3584 CG Utrecht, Netherlands; ^2^Immunology Platform, Danone Nutricia Research, Uppsalalaan 12, 3584 CT PO Box 80141, 3508 TC, Utrecht, Netherlands; ^3^Department of Immunotoxicology, Institute for Risk Assessment Sciences, Faculty of Veterinary Medicine, Utrecht University, Yalelaan 104, 3584 CM Utrecht, Netherlands

## Abstract

**Background:**

In previous studies, we showed that a fructo-oligosaccharide- (FOS-) supplemented diet enhanced oral immunotherapy (OIT) efficacy in a mouse model for cow's milk allergy. Fermentation of FOS by intestinal bacteria leads to production of short-chain fatty acids (SCFA) including butyrate.

**Aim:**

To investigate the contribution of butyrate in the enhanced efficacy of OIT + FOS.

**Methods:**

C3H/HeOuJ mice were sensitized and received OIT with or without FOS or butyrate supplementation. After treatment, whole blood was collected to conduct a basophil activation test (BAT) and allergen challenges were performed to measure acute allergic symptoms. CD4 + CD25 + regulatory T cells (Tregs) were isolated from treated mice or differentiated *in vitro* and used in a bone marrow-derived mast cell (BMMC) suppression assay. Cecum content was collected to analyze SCFA concentrations.

**Results:**

Allergen-induced basophil activation was reduced in OIT + butyrate samples compared to OIT. Accordingly, the acute allergic skin response and mast cell degranulation upon challenge were reduced in OIT + butyrate and OIT + FOS mice compared to sensitized controls. Butyrate was increased in the cecum content of OIT + FOS mice compared to OIT mice and sensitized controls. Treg-mediated BMMC suppression was enhanced after *in vivo* butyrate and FOS exposure in combination with OIT but with a more pronounced effect for butyrate.

**Conclusion:**

Butyrate supplementation enhanced OIT-induced desensitization of basophils and mast cells and Treg functionality. Only OIT + FOS treatment induced potential microbial alterations, shown by increased butyrate levels in cecum content. Both butyrate and FOS are promising candidates to improve OIT efficacy in human studies to treat food allergies.

## 1. Introduction

Population-based sampling of Australian one-year-old infants showed oral challenge-proven IgE-mediated food allergy in over 10% of the infants [1]. This high prevalence of food allergies among infants, in combination with an associated reduced growth and increased risk of asthma development later in life [2, 3], stresses the need for effective interventions. To date, food allergy management largely consists of allergen avoidance and administration of epinephrine in case of systemic anaphylaxis. Human trials with antigen-specific immunotherapy (AIT) to treat food allergies have shown promising results. However, safety and efficacy concerns have obstructed widespread clinical application [4, 5]. A recent meta-analysis confirmed that AIT leads to an increase in the tolerated dose in food allergic patients but also reported an increased risk of mild to severe adverse (systemic) reactions during therapy [6]. In addition, practical guidelines on AIT for the treatment of IgE-mediated food allergy have been prepared and published by the European Academy of Allergy and Clinical Immunology (EAACI) [7].

Oral immunotherapy (OIT) to treat cow's milk, peanut, and hen's egg allergies has been shown to reduce clinical symptoms upon food challenge but unsuccessfully maintained the protective state upon discontinuation of the therapy [8]. Effective desensitization of effector cells like mast cells and basophils in combination with active modulation of the adaptive immune response via antigen-presenting cells and T and B lymphocytes is key mechanisms in OIT [9]. The use of dietary adjuvants with immunomodulatory properties might open a new window of opportunities to improve the efficacy of OIT for food allergies.

Pre- and probiotics have been shown to promote oral tolerance and attenuate the allergic phenotype via the growth of beneficial microbes in the gut and the increased production of short-chain fatty acids (SCFA) [10, 11]. Coadministration of a probiotic during OIT in peanut allergic children induced suspected sustained unresponsiveness to a food challenge in 82.1% of the participants after 2–5 weeks without therapy [12]. Previous studies from our group have shown that dietary supplementation with fructo-oligosaccharides (FOS, prebiotics) during OIT improved the efficacy of the therapy in a murine cow's milk allergy model [13]. We observed a reduction in clinical symptoms upon food challenge, including reduced mucosal mast cell degranulation, and showed the involvement of Foxp3+ regulatory T cells (Tregs) in the protective effect induced by OIT + FOS [13]. In addition, the interaction of proteins and nondigestible oligosaccharides with intestinal epithelial cells (IEC) can induce release of soluble galectin-9, a glycan-recognizing protein involved in tolerance induction and direct suppression of IgE-mediated mast cell degranulation [14]. A significant increase in serum galectin-9 levels was observed after OIT + FOS treatment in cow's milk allergic mice [13].

Fermentation of nondigestible oligosaccharides and proteins by commensal microbes, present in the colon and cecum, leads to the formation of SCFA. Specific bacterial groups are responsible for the production of butyrate from acetyl-CoA and butyryl-CoA, propionate from propionyl-CoA, and acetate from acetyl-CoA [15]. After absorption into colonic or cecal epithelial cells via diverse mechanisms, SCFA enter the circulation and modulate metabolic and immune processes in peripheral tissues [16]. Via the inhibition of histone deacetylases (HDAC) and activation of G protein-coupled receptors (GPCR), e.g., GPR41, GPR43, and GPR109a, on epithelial and immune cells, SCFA can alter gene expression and inflammatory responses [17].

To gain more insight into the role of butyrate in the allergy protective effect induced by OIT and FOS supplementation, we administered butyrate directly to cow's milk allergic mice during OIT and evaluated the allergic response to food challenges.

## 2. Materials and Methods

### 2.1. Mice

**Figure 1:**
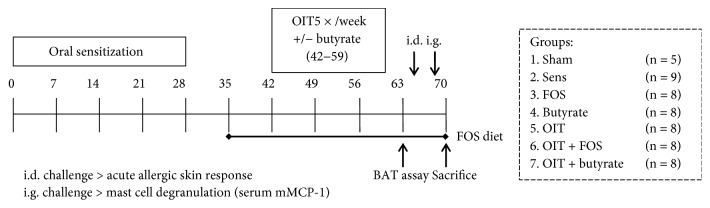
Experimental timeline and groups. Female C3H/HeOuJ mice were grouped as depicted: sham, sham-sensitized control; sens, whey-sensitized control; FOS, FOS-supplemented group; butyrate, butyrate-supplemented group; OIT, OIT group; OIT + FOS, OIT with FOS supplementation group; and OIT + butyrate, OIT with butyrate supplementation group. All mice were fed the AIN-93G control diet upon arrival and during i.g. sensitization with whey (20 mg in 0.5 ml PBS) and cholera toxin (15 *μ*g in 0.5 ml PBS) on days 0, 7, 14, 21, and 28. The FOS-supplemented diet was provided from D35 to the end of the protocol in the FOS and OIT + FOS groups. OIT with 10 mg whey in 0.5 ml PBS was given 5×/week for 3 weeks (D42–59). Sodium butyrate was coadministered during OIT (0.6 g/kg bodyweight/day) based on mean bodyweight per group. At D63, whole blood samples were collected via cheek puncture to perform a BAT and an i.d. challenge (D64, 10 *μ*g whey in 20 *μ*l PBS/ear) and i.g. challenge (D69, 50 mg whey in 0.5 ml PBS) were conducted to measure the acute allergic skin response and mucosal mast cell degranulation (mMCP-1), respectively. At D70, all mice were sacrificed and blood and organs were collected. OIT: oral immunotherapy; FOS: fructo-oligosaccharides; CT: cholera toxin; i.d.: intradermal; i.g.: intragastric; BAT: basophil activation test; mMCP-1: mucosal mast cell protease-1.

Six-week-old female specific-pathogen free C3H/HeOuJ mice (*n* = 54) were purchased (Charles River Laboratories, Erkrath, Germany) and randomly allocated to the control and experimental groups: sham, sham-sensitized control (*n* = 5); sens, whey-sensitized control (*n* = 9); FOS, FOS-supplemented group (*n* = 8); butyrate, butyrate-supplemented group (*n* = 8); OIT, OIT group (*n* = 8); OIT + FOS, OIT with FOS supplementation group (*n* = 8); and OIT + butyrate, OIT with butyrate supplementation group (*n* = 8) (as depicted in Figure 1). All mice were housed in filter-topped makrolon cages (one cage/group) on a 12 h light/dark cycle with unlimited access to food and water at the animal facility of Utrecht University and were acclimatized for 6 days. All experimental procedures were approved by the Ethical Committee of Animal Research of Utrecht University and complied with the principles of good laboratory animal care as stated by the European Directive for the protection of animals used for scientific purposes.

### 2.2. Control Diet and FOS-Supplemented Diet

All animals were fed the AIN-93G control diet during acclimatization and oral sensitization (D0 to D35). The FOS-supplemented diet was provided from D35 to the end of the protocol in the FOS and OIT + FOS groups (Figure 1). Shortly, a specific mixture of plant-derived short-chain FOS (scFOS: oligofructose, Raftilose P95, degree of polymerization (DP) < 6) and long-chain FOS (lcFOS: long-chain inulin, Raftiline HP, average DP of 23 or higher with <1% DP of 5 or lower) was provided by Orafti (Wijchen, the Netherlands) and added to the base recipe of the cow's milk protein-free AIN-93G diet (scFOS/lcFOS, 9 : 1, 1%, w/w, Ssniff Spezialdiäten GmbH, Soest, Germany) [13].

### 2.3. Experimental Animal Procedures

All mice were intragastrically (i.g.) sensitized to the cow's milk protein whey (DMV International, Veghel, the Netherlands) dissolved in PBS (20 mg whey in 0.5 ml PBS, Lonza, Verviers, Belgium) with cholera toxin (CT, 15 *μ*g CT in 0.5 ml, List Biological Laboratories Inc., Campbell, CA, USA) to induce food allergy or were sham-sensitized with PBS and CT alone on D0, 7, 14, 21, and 28 (Figure 1). OIT consisted of 10 mg whey in 0.5 ml PBS and was provided per oral gavage from D42–D59 (5×/week, for 3 weeks). The butyrate and OIT + butyrate mice were weighted prior to D42, D49, and D56 and received 0.6 g/kg bodyweight/day sodium butyrate (Sigma-Aldrich, Zwijndrecht, the Netherlands) dissolved in PBS with or without whey based on mean bodyweight of the group. To assess allergic status after treatment, all mice received an intradermal (i.d.) challenge in both ear pinnae (10 *μ*g whey in 20 *μ*l PBS/ear) to measure the acute allergic skin response and symptoms of anaphylaxis on experimental day 64. Mean basal ear thickness in *μ*m (using a digital micrometer, Mitutoyo, Veenendaal, the Netherlands) was subtracted from mean ear thickness 1 h postchallenge (in duplicate, in both ears, blinded measurement) to calculate Δ ear swelling (i.e., the acute allergic skin response) per mouse. To perform the i.d. injection and both ear measurements, all mice were anesthetized twice using inhalation of isoflurane. To assess mucosal mast cell degranulation, all mice were i.g. challenged with 50 mg whey in 0.5 ml PBS on experimental day 69. Serum samples were collected via cheek puncture 30 min after i.g. provocation to measure mucosal mast cell protease-1 (mMCP-1) concentrations. The mice were sacrificed at D70 to collect blood and organs.

### 2.4. Basophil Activation Test

Whole blood samples from all control and experimental groups were collected via cheek puncture at D63 to conduct a basophil activation test (BAT) according to the method described by Torrero et al. [18]. Briefly, whole blood was incubated (1.5 h at 37°C) with RPMI-1640 medium (Lonza), *α*IgE (125 ng/ml, eBioscience, Breda, the Netherlands), or whey (20 *μ*g/ml, DMV International) to activate basophils. After red blood cell lysis (Whole Blood Lysing Reagents, Beckman Coulter, Fullerton, CA, USA), cells were stained with anti-IgE-FITC, anti-CD49b-APC, anti-CD4-PE, and anti-B220-PE (eBioscience) to select the basophil population while excluding T and B cells. Median fluorescence intensity (MFI) of activation marker CD200R-PerCp-eFluor 710 was determined with flow cytometry using a FACS Canto II (BD Biosciences, Alphen a/d Rijn, the Netherlands).

### 2.5. ELISA

Serum samples collected prior to sacrifice were stored at −20°C until analysis of whey-specific antibodies (IgE, IgA, IgG1, and IgG2a), mMCP-1, and galectin-9. Culture supernatants from bone marrow-derived mast cell (BMMC) assays were collected and stored at −20°C to measure IL-6 and IL-13 concentrations. All procedures were conducted as described elsewhere [13].

### 2.6. SCFA Analysis in Cecum Supernatant

To determine SCFA concentrations, cecum content was collected and stored at −80°C until further processing according to the method described previously [19]. Cecum supernatant was analyzed using a Shimadzu GC2010 gas chromatograph (Shimadzu Corporation, Kyoto, Japan).

### 2.7. Tregs and BMMC Suppression Assays

BMMC were cultured from naïve female C3H/HeOuJ mice in RPMI-1640 medium (Lonza) supplemented with 10% FCS, 26 mM Hepes, 0.12 mM MEM nonessential amino acids, 2.4 mM Glutamax, 1.2 mM sodium pyruvate (all from Gibco Thermo Fisher Scientific, Waltham, MA, USA), penicillin-streptomycin (100 U/ml–100 *μ*g/ml, Sigma-Aldrich), and IL-3 and stem cell factor (SCF, both 10 ng/ml, Prospec, Ness-Ziona, Israel) at 37°C with 5% CO_2_.

CD4 + CD25 + Tregs were purified from pooled whole spleen suspensions derived from all control and experimental groups of mice in a follow-up experiment according to the manufacturer's instructions (Miltenyi Biotec, Leiden, the Netherlands) and were cocultured in a 1 : 1 ratio with BMMC sensitized with anti-DNP-IgE (dinitrophenol) according to the method described elsewhere [20]. Subsequently, BMMC were activated with 25 ng/ml DNP-HSA (DNP hapten conjugated to human serum albumin) and release of *β*-hexosaminidase (*β*-hex) was measured. Activated BMMC were incubated in fresh medium for 24 h to collect culture supernatant for cytokine analysis.

*In vitro* Treg induction from naïve CD4+CD25− splenocytes was conducted according to the method described previously [21]. 125 *μ*M sodium butyrate (Sigma-Aldrich) and 0.05% scFOS/lcFOS (9 : 1, Orafti) were added to the culture medium. After 6 days of incubation at 37°C with 5% CO_2_ in the presence of IL-2 (20 ng/ml) and TGF*β* (5 ng/ml), cells were harvested and cocultured with BMMC sensitized with anti-DNP-IgE as described earlier. Beta-hex release was measured upon BMMC activation using DNP-HSA. An aliquot of cells was stained for anti-CD4-FITC, anti-CD25-PE, and anti-Foxp3-APC (eBioscience) and analyzed using flow cytometry.

### 2.8. Data Analysis and Statistics

**Figure 2:**
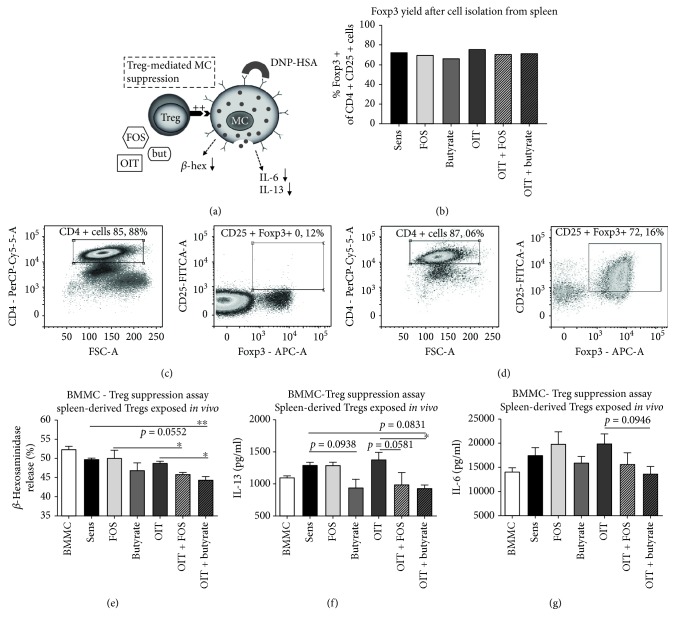
Enhanced Treg-mediated BMMC suppression after exposure to OIT and butyrate supplementation *in vivo*. CD4+ cells derived from pooled spleen suspensions were separated based on CD25 positivity. (a) Schematic representation of BMMC-Treg suppression assay. (b) CD4+CD25+ cells were verified for Foxp3 expression with flow cytometry and showed ±70% positivity in all groups. (c) CD4+CD25− cells and (d) CD4+CD25+ cells after MACS separation. Subsequently, CD4+CD25+ cells were cocultured with BMMC sensitized with anti-DNP-IgE. (e) Reduced release of *β*-hex upon BMMC activation with 25 ng/ml DNP-HSA was observed in the presence of Tregs derived from OIT + butyrate mice compared to Tregs derived from OIT and sensitized control mice. Additional 24 h incubation of BMMC in fresh culture medium indicated (f) reduced production of IL-13 after coculture with OIT + butyrate-Tregs compared to OIT-Tregs and (g) no differences in IL-6 release by activated BMMC. Data are represented as mean ± SD in (e–g) duplicate measurements. Statistical analysis was performed using one-way ANOVA and Bonferroni's post hoc test to compare preselected combinations. ^∗^*p* < 0.05, ^∗∗^*p* < 0.01. FSC-A: forward scatter-area; OIT: oral immunotherapy; FOS: fructo-oligosaccharides; but: butyrate; Treg: regulatory T cell; MC: mast cell; BMMC: bone marrow-derived mast cell; *β*-hex: *β*-hexosaminidase; DNP-HSA: dinitrophenol hapten conjugated to human serum albumin.

**Figure 3:**
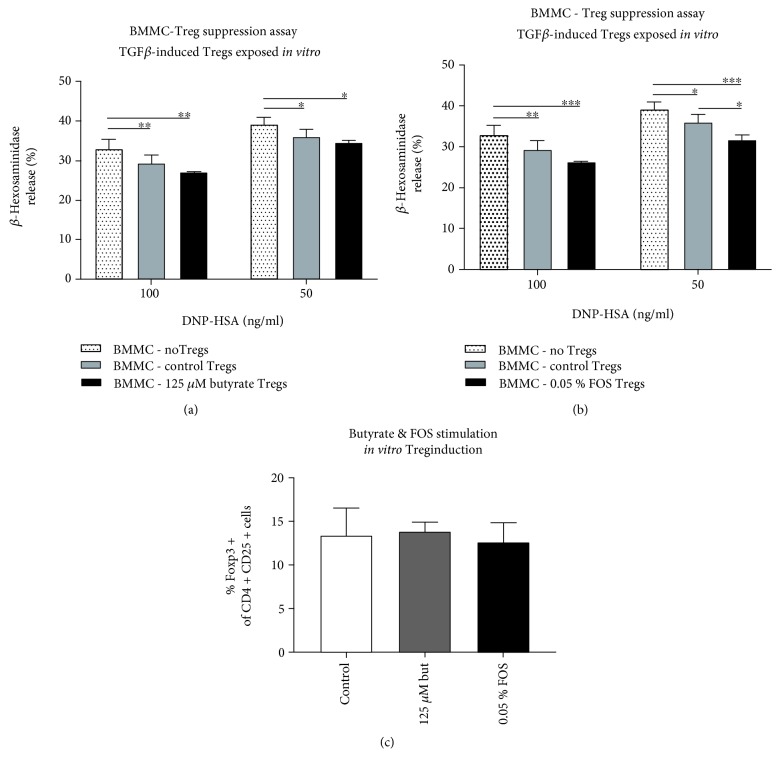
Enhanced Treg-mediated BMMC suppression after *in vitro* exposure to FOS. TGF*β* and IL-2-mediated induction of Tregs from naïve CD4+CD25− splenocytes resulted in functional cells, shown by reduced BMMC activation. (a) Exposure to butyrate (125 *μ*M) did not enhance Treg-mediated suppression of BMMC. (b) Exposure to FOS (0.05%) significantly improved Treg-mediated BMMC suppression upon activation with 50 ng/ml DNP-HSA compared to control Tregs. (c) No differences in Foxp3 yield were observed in the control, butyrate, or FOS condition. Data are depicted as mean ± SD, (a, b) representative experiment, duplicate measurements per concentration DNP-HSA. (c) Mean of 3 independent experiments, duplicate measurements. Statistical analysis was performed using two-way ANOVA for nonrepeated measures (a, b) and one-way ANOVA (c) with Bonferroni's post hoc test to compare preselected combinations. ^∗^*p* < 0.05, ^∗∗^*p* < 0.01, ^∗∗∗^*p* < 0.001. BMMC: bone marrow-derived mast cells; Treg: regulatory T cells; FOS: fructo-oligosaccharides; DNP-HSA: dinitrophenol hapten conjugated to human serum albumin; but: butyrate.

Data were presented as mean ± SEM and were analyzed using GraphPad Prism software version 7 (GraphPad software, La Jolla, CA, USA). Beta-hex data in Figures 2(e) and 3 and ELISA data in Figures 2(f) and 2(g) are depicted as mean ± SD. We used one-way ANOVA and Bonferroni's post hoc test to compare sham with sens; sens with FOS, butyrate, OIT, OIT + FOS, and OIT + butyrate; FOS with OIT + FOS; butyrate with OIT + butyrate; and OIT with OIT + FOS and OIT + butyrate. Whey-specific antibody data were log-transformed prior to testing and the median is depicted per group. Beta-hex data in Figure 3 were analyzed using a two-way ANOVA for nonrepeated measures. Calculated *p* values were corrected for the number of comparisons and were considered statistically significant when *p* < 0.05.

## 3. Results

### 3.1. Butyrate Supplementation Supported OIT Efficacy Shown by Reduced Effector Cell Activation upon Challenge

**Figure 4:**
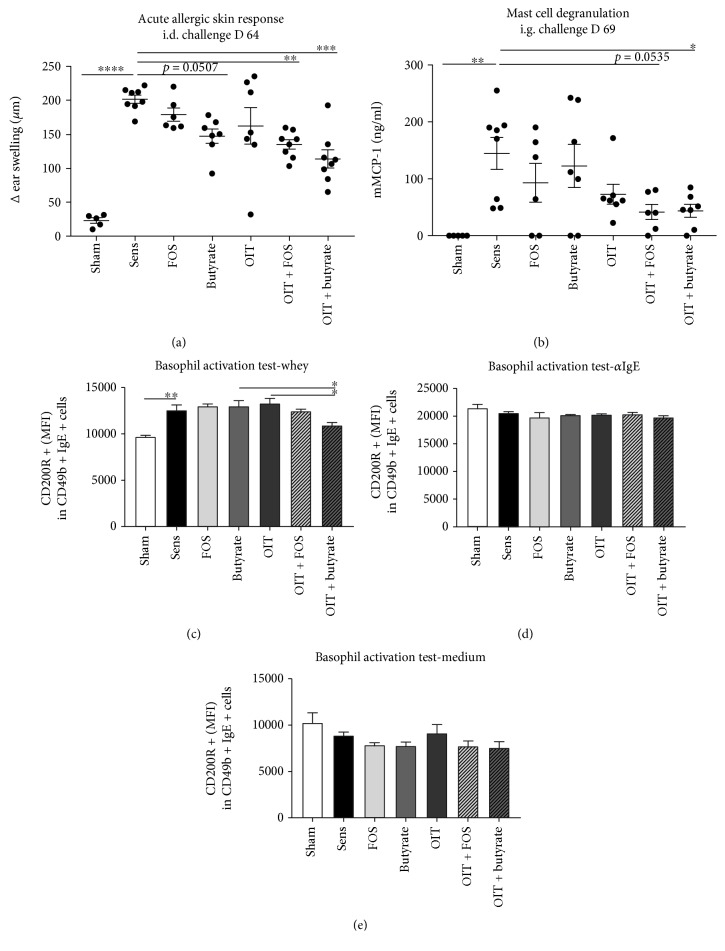
Reduced activation of allergic effector cells upon challenge after OIT and butyrate or FOS supplementation. (a) Reduced acute allergic skin response (Δ ear swelling 1 h after i.d. injection with whey) in OIT + FOS and OIT + butyrate mice compared to sensitized controls. (b) Reduced mucosal mast cell degranulation (serum mMCP-1 concentration) in OIT + butyrate mice compared to sensitized controls. Decreased MFI of CD200R in basophils activated with (c) whey in OIT + butyrate blood samples compared to OIT and butyrate samples. No differences in MFI observed after basophil stimulation with (d) *α*IgE and (e) medium. Data are represented as mean ± SEM, *n* = 5–9/group (a, b) and *n* = 3–5/group (c, d) (whole blood samples were pooled per 2 mice). Statistical analysis was performed using one-way ANOVA and Bonferroni's post hoc test to compare preselected combinations. ^∗^*p* < 0.05, ^∗∗^*p* < 0.01, ^∗∗∗^*p* < 0.001, ^∗∗∗∗^*p* < 0.0001. i.d.: intradermal; i.g.: intragastric; mMCP-1: mucosal mast cell protease-1; MFI: median fluorescence intensity.

The sensitized control group showed increased acute allergic skin responses (i.e., magnitude of the ear swelling response) and increased mucosal mast cell degranulation (i.e., serum mMCP-1) after challenge compared to the sham-sensitized control group (Figures 4(a) and 4(b)). OIT + butyrate reduced the acute allergic skin response and mucosal mast cell degranulation compared to sensitized controls (Figures 4(a) and 4(b)). A trend (*p* = 0.0507) toward a reduction in ear swelling was observed in the animals which were only exposed to oral butyrate supplementation (Figure 4(a)). In addition, basophils derived from whole blood of OIT + butyrate mice showed reduced expression of activation-associated receptor CD200R upon antigen-specific stimulation compared to OIT- and butyrate-derived basophils (Figure 4(c)). No differences between the groups were observed after stimulation with *α*IgE and medium, indicating modulation of the antigen-specific basophil response (Figures 4(d) and 4(e)). In accordance with previous results [13], OIT + FOS effectively reduced the acute allergic skin response (Figure 4(a)) and a trend (*p* = 0.0535) toward a reduction in mucosal mast cell degranulation was observed compared to sensitized controls (Figure 4(b)). Despite the observed reduction in the acute allergic skin responses in OIT + butyrate and OIT + FOS mice, no protection against symptoms of anaphylaxis, e.g., body temperature drop, induced by the i.d. challenge was observed (data not shown).

### 3.2. OIT Influenced Whey-Specific IgE Levels in Serum

**Figure 5:**
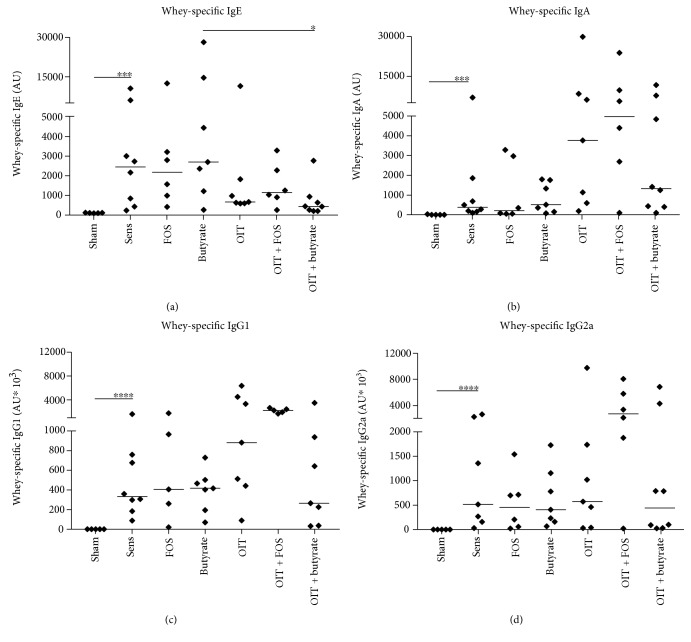
OIT influenced whey-specific IgE responses in mice. Whey-specific antibodies were measured by means of ELISA in serum collected at D70. (a) Whey-specific IgE, (b) whey-specific IgA, (c) whey-specific IgG1, and (d) whey-specific IgG2a levels were increased in whey-sensitized control mice compared to sham-sensitized controls. OIT decreased whey-specific IgE independent of the dietary interventions. OIT + FOS had the most pronounced effect on whey-specific IgA, IgG1, and IgG2a. Data are depicted as individual data points with the median per group, *n* = 5–8/group. Statistical analysis was performed by log-transforming the data followed by a one-way ANOVA with Bonferroni's post hoc test to compare preselected combinations. ^∗^*p* < 0.05, ^∗∗∗^*p* < 0.001, ^∗∗∗∗^*p* < 0.0001. AU: arbitrary units.

Levels of whey-specific IgE, IgA, IgG1, and IgG2a in serum collected at D70 were increased in the sensitized controls compared to the sham-sensitized controls (Figure 5(a)–5(d)). Except the whey-specific IgE levels which were lowered in the OIT + butyrate group compared to butyrate (Figure 5(a)), no significant differences in whey-specific antibody levels were found between the control and experimental groups. However, in accordance with previous results [22], the level of whey-specific IgE was lower in OIT-exposed animals. No additional effect of the dietary intervention with either FOS or butyrate was observed in the current study with respect to whey-specific IgE (Figure 5(a)). Whey-specific IgA, IgG1, and IgG2a levels were higher in the combination group OIT + FOS (Figure 5(b)–5(d)).

### 3.3. OIT + FOS Increased Local Butyrate Levels

**Figure 6:**
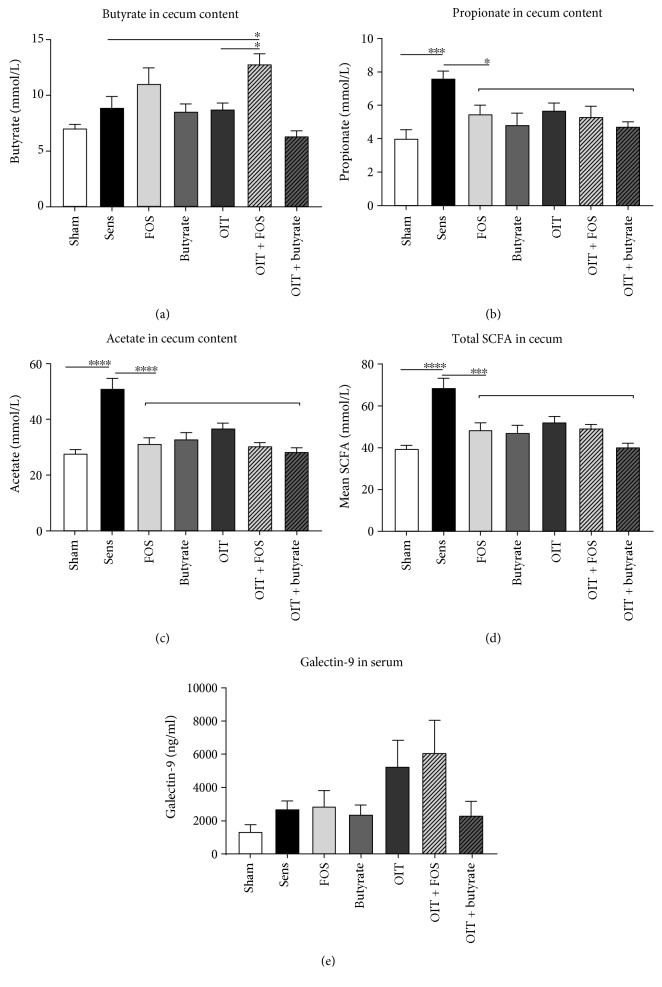
Increased butyrate concentrations in cecum content of OIT + FOS mice. SCFA analysis in cecum content (upon local fermentation) indicated that (a) butyrate levels were increased in OIT + FOS mice compared to OIT mice and sensitized controls. (b) Propionate and (c) acetate levels in cecum content were increased in sensitized controls compared to sham-sensitized controls. (d) Mean total SCFA levels were increased in the sensitized controls correspondingly. (e) Serum galectin-9 concentrations measured by means of ELISA. Data are represented as mean ± SEM, *n* = 5–8/group. Statistical analysis was performed using one-way ANOVA and Bonferroni's post hoc test to compare preselected combinations. ^∗^*p* < 0.05, ^∗∗∗^*p* < 0.001, ^∗∗∗∗^*p* < 0.0001. SCFA: short-chain fatty acids. The horizontal line drawn above the groups FOS, butyrate, OIT, OIT + FOS, and OIT + butyrate (b–d) indicates that all groups differ significantly from the sensitized control group with ^∗^*p* < 0.05 (b), ^∗∗∗∗^*p* < 0.0001 (c), and ^∗∗∗^*p* < 0.001 (d).

OIT + FOS increased levels of butyrate in cecum content compared to the sensitized control and the OIT groups which were fed the control diet (Figure 6(a)). No increase in butyrate in the cecum content was observed in butyrate and OIT + butyrate mice, suggesting systemic uptake of orally administered butyrate. Oral sensitization against whey increased propionate and acetate concentrations as observed in cecum content of the whey-sensitized controls compared to the sham-sensitized controls (Figures 6(b) and 6(c)). Total SCFA levels in cecum content of sensitized control mice were increased accordingly (Figure 6(d)). In the current study, no significant differences in serum galectin-9 concentrations were observed between the groups (Figure 6(e)).

### 3.4. Tregs Derived from OIT + Butyrate Mice Showed Enhanced Suppression of BMMC Responses Compared to OIT Mice and Sensitized Control Mice

A schematic representation of the *ex vivo* Treg-BMMC suppression assay is shown in Figure 2(a). Spleen-derived CD4+CD25+ cells were isolated and verified for Foxp3 expression (Figure 2(b)–2(d), approximately 70% positivity) and cocultured with anti-DNP-IgE-sensitized naïve BMMC. Tregs derived from OIT + butyrate mice reduced *β*-hex release upon BMMC activation with DNP-HSA compared to Tregs derived from OIT mice and sensitized control mice (Figure 2(e)). In addition, a significant reduction in IL-13 release by BMMC was only observed with OIT + butyrate-derived Tregs compared to OIT Tregs (Figure 2(f)). A trend toward a reduction in *β*-hex release was observed with OIT + FOS Tregs compared to sensitized control Tregs (*p* = 0.0552, Figure 2(e)) and a trend toward a reduction in IL-13 release by BMMC was observed compared to OIT (*p* = 0.0581, Figure 2(f)). OIT + FOS-derived Tregs only significantly reduced *β*-hex release compared to Tregs from FOS-supplemented mice (Figure 2(e)). No difference in the release of IL-6 by activated BMMC was observed in the Treg-BMMC cocultures, except the trend toward a reduction in IL-6 release observed with OIT + butyrate Tregs compared to OIT (*p* = 0.0946, Figure 2(g)).

### 3.5. FOS Exposure during In Vitro Treg Induction Enhanced BMMC Suppression

*In vitro* stimulation of naïve spleen-derived CD4+CD25− cells with TGF*β* and IL-2 led to differentiation into Foxp3+ Tregs with functional suppressive capacities shown by reduced BMMC activation (Figures 3(a) and 3(b)). The percentage of Foxp3+ cells after 6 days of stimulation was similar in all conditions (Figure 3(c)). Although exposure to butyrate during Treg induction could not further improve the suppressive action of the Tregs toward BMMC (Figure 3(a)), direct exposure to FOS did enhance Treg-mediated BMMC suppression (Figure 3(b)). In particular, BMMC activation with 50 ng/ml antigen was significantly reduced in the presence of FOS-exposed Tregs compared to control Tregs (Figure 3(b)).

## 4. Discussion

The presented data indicate that butyrate supplementation is as effective as FOS supplementation in supporting OIT-induced desensitization in a murine cow's milk allergy model, shown by reduced responsiveness of effector cells to antigen-specific challenges conducted *in vivo*. The observed alterations in specific basophil responses and the suppressive capacity of Tregs toward cultured mast cells were more pronounced after OIT + butyrate treatment. Interestingly, local butyrate levels were only elevated after OIT + FOS treatment, suggesting specific changes in the activity of the microbiota in the gastrointestinal tract.

In the current study, mice receiving OIT alone showed no reduction in acute allergic symptoms upon antigen-specific challenge. It has been described that desensitization of effector cells like mast cells and basophils is one of the earliest events observed in antigen-specific immunotherapy [23]. Despite the presence of (high levels of) specific IgE, mast cells and basophils show reduced degranulation capacity followed by suppressed systemic anaphylaxis symptoms [23]. Cross-linking of surface-bound IgE by intact allergens simultaneously upregulates expression of the inhibitory histamine receptor 2 on basophils, thereby providing a control system for cell activation [24]. The combination therapies OIT + butyrate and OIT + FOS did effectively induce desensitization of mast cells as observed at two distinct sites in the mice: connective tissue-mast cells residing in the skin of the ear (upon i.d. challenge) and mucosal-mast cells residing in the gastrointestinal tract (upon i.g. challenge). Systemically available butyrate might have affected mast cell functionality at both sites, since it has been shown *in vitro* that butyrate reduced proliferation and cytokine production by mast cells via HDAC inhibition [25].

Firstly, as observed in the current study, oral butyrate supplementation alone did not lead to a significant reduction in mast cell degranulation provoked by food challenge compared to the sensitized controls. Moreover, *ex vivo* whey-specific basophil activation in whole blood samples was only reduced after providing OIT and butyrate supplementation simultaneously. Thereby, it was demonstrated that both butyrate and whey are key components in the desensitization process of basophils in cow's milk allergic mice. Secondly, a direct effect of FOS supplementation on *in vivo* mast cell degranulation was not observed; however, it has been shown *in vitro* that specific human milk oligosaccharides (HMOS) are able to directly inhibit IgE-mediated mast cell activation but only at a high concentration of 1 mg/ml [20]. Previous studies showed epithelial transfer of nondigestible oligosaccharides *in vitro* [26] and confirmed systemic availability of HMOS in breast-fed infants [27]. It needs to be elucidated whether physiologically relevant FOS concentrations reach mast cells residing in the mucosal or connective tissues and whether FOS directly contribute to the observed reduced degranulation response.

The involvement of CD4+CD25+Foxp3+ Tregs in controlling the allergic response has been described earlier [28]. Here, we showed enhanced suppression of BMMC responses (*β*-hex and IL-13) upon Fc*ε*RI-mediated activation by Tregs derived from OIT + butyrate mice and a tendency in OIT + FOS mice compared to sensitized controls. Moreover, the suppressive capacity of Tregs derived from OIT + butyrate mice was improved compared to OIT alone. Enhanced Treg functionality was previously observed in an adoptive transfer experiment using OIT + FOS donor mice: *ex vivo* Treg-depleted cell fractions could not control mucosal mast cell degranulation upon allergen challenge in recipients [13]. The data derived from the *in vitro* experiments indicated that FOS exposure directly affected Treg functionality in a butyrate-independent manner.

As described previously, oral butyrate supplementation exerts anti-inflammatory effects in preclinical models of colitis and liver disease [29, 30]. In addition, butyrate enemas were shown to ameliorate symptoms in ulcerative colitis patients [31]. In the context of food allergy prevention, protection against anaphylaxis and a reduction in total IgE were observed in peanut allergic mice after oral supplementation with butyrate or acetate during sensitization, mimicking the beneficial effects mediated by high fiber intake [32]. In the current experiment, butyrate was either directly available per oral gavage or indirectly available after FOS fermentation. The effect of (high) butyrate exposure on composition and activity of the microbiome is poorly defined. Butyrate treatment in mice suffering from enteritis affected the abundance of specific bacterial species in addition to reduced intestinal inflammation [33]. Clinical trials on the application of SCFA to treat colonic inflammatory disorders reported varying success rates and do not include microbiome data [34]. Administration of FOS during OIT induced specific alterations in microbial communities present in the gastrointestinal tract, reflected by increased butyrate levels in cecum content of OIT + FOS mice. In humans, FOS supplementation in combination with *Bifidobacterium breve* M16V (synbiotics) improved microbiota composition in non-IgE-mediated cow's milk allergic infants that were fed an amino acid-based infant formula [35]. In addition to the microbiota effects, direct interaction of nondigestible oligosaccharides via glycan receptors with IEC and immune cells resident in the lamina propria of the gut contributes to orchestration of the mucosal immune response [36]. *In vitro* stimulation of IEC with nondigestible oligosaccharides in combination with bacterial DNA or a TLR-9 ligand leads to the release of galectin-9. IEC-derived galectin-9 induced IFN*γ* secretion by activated PBMC and stimulated proliferation of Th1 cells and Tregs [37]. Galectin-9 present in serum derived from whey-sensitized mice supplemented with a synbiotic concept containing nondigestible oligosaccharides suppressed *in vitro* degranulation of RBL-2H3 cells and was associated with reduced allergic symptoms [38]. However, the contribution of galectin-9 in effector cell suppression remains elusive in this experimental model, since no statistically significant differences in serum galectin-9 levels were observed in the current study.

The used butyrate dose was previously shown to be safe in mice [29] and no signs of (mild) toxicity were detected. However, butyrate administration should be performed with caution, since previous studies reported cases of hypokalemia, nausea, and seizure after intravenous injection [39, 40]. FOS supplementation has been considered to be safe and is used as a supplement in infant formula for cow's milk allergic infants [41].

## 5. Conclusion

Butyrate supplementation enhanced desensitization of effector cells induced by OIT in cow's milk allergic mice. The improvement of OIT efficacy was previously only described for FOS supplementation. We showed effective reduction of mast cell activation upon *in vivo* challenge and basophil activation upon *ex vivo* challenge and enhanced suppressive activity of OIT + butyrate-derived Tregs. Fermentation-derived SCFA levels in cecum content suggest alterations in microbial communities and/or activity followed by FOS supplementation during OIT. In addition, FOS stimulation directly enhanced the suppressive capacity of *in vitro* differentiated Tregs toward cultured mast cells, in a butyrate-independent manner. Therefore, additional studies should clarify the role of FOS during allergy protection. Moreover, the contribution of butyrate in FOS-mediated effects needs to be confirmed with a more mechanistic approach. However, both FOS and butyrate are promising candidates to improve OIT efficacy in future human food allergy trials.

## Data Availability

The data used to support the findings of this study are available from the corresponding author upon request.
